# Capecitabine plus Oxaliplatin as a Second-Line Therapy for Advanced Biliary Tract Cancers: A Multicenter, Open-Label, Phase II Trial

**DOI:** 10.7150/jca.37610

**Published:** 2019-10-15

**Authors:** Seung Tae Kim, Sung Yong Oh, Jeeyun Lee, Jung Hun Kang, Hyun Woo Lee, Myung Ah Lee, Byeong Seok Sohn, Ji Hyong Hong, Young Suk Park, Joon Oh Park, Ho Yeong Lim

**Affiliations:** 1 Department of Medicine, Sungkyunkwan University School of Medicine, Samsung Medical Centre, Seoul, South Korea;; 2 Department of Medicine, Dong-A University School of Medicine, Busan, South Korea;; 3 Department of Medicine, Gyeongsang National University Hospital, Jinju, South Korea;; 4 Department of Medicine, Ajou University School of Medicine, Suwon, South Korea;; 5 Department of Medicine, Seoul St Mary's Hospital, Catholic University, Seoul, South Korea;; 6 Department of Medicine, Sanggye Paik Hospital, Inje University College of Medicine, Seoul, South Korea;; 7 Department of Medicine, Incheon St Mary's Hospital, Catholic University, Incheon, South Korea.

**Keywords:** biliary tract cancer, capecitabine, oxaliplatin

## Abstract

**Background:** Although biliary tract cancer (BTC) has a very aggressive nature, some patients maintain a relatively good performance status after failure with first-line treatment of gemcitabine plus cisplatin (GC). Thus, tolerable, feasible, and useful second-line treatments are needed for these patients. We investigated the efficacy of capecitabine plus oxaliplatin (XELOX) as a second-line therapy for patients with advanced BTC who failed first-line GC treatment.

**Methods:** In this prospective, phase II trial, we investigated XELOX (capecitabine 1,000 mg/m^2^ twice daily on days 1-14 and oxaliplatin 130 mg/m^2^ on day 1) as a second-line treatment, given every 3 weeks, totaling 8 cycles in patients with metastatic BTC who failed first-line GC treatment. The primary outcome was progression-free survival (PFS).

**Results:** From December 2015 to November 2016, 50 patients with metastatic intrahepatic or extrahepatic cholangiocarcinoma or gall bladder (GB) cancer were enrolled. The regimen was well tolerated. Toxicities mainly consisted of grade 1 or 2 events, and thrombocytopenia and neuropathy had the highest incidence. In intent-to-treat analysis, one complete response (CR) and six partial responses (PRs) were recorded with XELOX treatment. The overall response rate and the disease control rate from the intent-to-treat analysis were 14% and 52%, respectively. With a median follow-up of 15.6 months, PFS after XELOX was a median of 15.4 weeks (95% CI, 8.5-22.3). This PFS value supported the statistical hypothesis of this study. The median overall survival was 32.7 weeks (95% CI, 21.4-43.9).

**Conclusion:** This phase II trial showed that XELOX treatment was efficacious and had a tolerable toxicity profile in patients with advanced BTC who failed first-line treatment of gemcitabine and cisplatin.

## Introduction

Biliary tract cancers (BTCs) are a heterogeneous disease group that includes cholangiocarcinoma and gallbladder (GB) cancer.^1^ BTCs are relatively rare tumors, accounting for 3% of all gastrointestinal tumors.^2^ However, in Korea, BTCs are not uncommon, as approximately 3,500 patients are newly diagnosed with the disease each year, and BTCs comprise 6% of cancer deaths.^3^ BTCs do not present specific clinical symptoms until an advanced stage; thus, most patients are diagnosed with an advanced stage of the disease.^4^ Recently, the treatment outcomes of advanced BTC patients have improved, and the median overall survival has reached almost one year. Gemcitabine and cisplatin (GC) combination therapy is the standard of care for advanced BTC.^5^ However, most patients with advanced BTC receiving GC as first-line therapy experience disease progression. Fortunately, approximately half of patients still have a good performance status after failure of frontline GC; these patients are candidates for subsequent second-line chemotherapy.^6^ However, appropriate second-line therapy has not yet been established for advanced BTCs.

One of the most widely used cytotoxic agents for BTCs is 5-fluorouracil (5-FU), either alone or in combination with other drugs.^7^-^10^ Capecitabine (Xeloda; F. Hoffmann-La Roche, Ltd.) is an oral fluoropyrimidine prodrug with preferential conversion to 5-FU in tumor tissue.^11^ Oxaliplatin is a third-generation platinum drug, with activity and toxicity profiles that differ from those of other platinum derivatives, including cisplatin and carboplatin. Oxaliplatin is used in clinical practice instead of cisplatin^12^ and has clinical activity and tolerable safety with capecitabine or 5-FU in many cancer types.^13^-^18^ Capecitabine plus oxaliplatin (XELOX) is used as the standard treatment for gastric cancer, colorectal cancer, and other gastrointestinal tumors.^14^,^19^,^20^

This phase II trial primarily aimed to evaluate the effect of XELOX on progression-free survival (PFS) in patients with advanced BTC.

## Methods

### Study Design

This study was a multicenter, open-label, phase II trial of XELOX combination therapy in patients with metastatic adenocarcinoma of the biliary tract who experienced failure of GC with first-line therapy. The primary objective of the study was to evaluate PFS, and secondary objectives were assessment of tumor response rates, toxicity, and overall survival. The institutional review board or ethics committee of each study site reviewed and approved the study protocol. All patients provided written informed consent according to institutional guidelines before study entry. This study was conducted in accordance with the Declaration of Helsinki.

### Participants

Patients with recurrent or metastatic disease who had histologically or cytologically confirmed adenocarcinoma originating from the biliary tract, which consists of the intrahepatic and extrahepatic bile ducts and the gallbladder, were eligible. All patients had experienced disease progression after first-line GC therapy. Adequate organ function, measurable or evaluable disease per the Response Evaluation Criteria in Solid Tumors (RECIST) 1.1, and an Eastern Cooperative Oncology Group (ECOG) performance status of 0-2 was defined by laboratory and imaging tests and physical examinations. Patients were excluded if they had ampulla of Vater cancer, locally advanced BTC, active central nervous system metastasis or infection, additional malignancy, or clinically significant comorbidities. Patients who previously had undergone two or more palliative chemotherapy treatments for BCT or had previous exposure to oxaliplatin or 5-FU including capecitabine were also excluded.

### Treatment and Dose Modification

In this study, we followed the dose and schedule of chemotherapy from current standards for XELOX. A total of 1,000 mg/m^2^ capecitabine was administered orally twice daily (bid) on days 1-14 and 130 mg/m^2^ oxaliplatin as a 120-min infusion on day 1.^21^ Treatment was repeated every 3 weeks in both groups, for a total of 8 cycles, and was discontinued in cases of disease progression, unacceptable toxicities, or consent withdrawal.

Dose adjustments were made based on the worst toxicity detected during the preceding cycle, as defined per protocol. Any patient who required a dose reduction for subsequent cycles continued to receive a reduced dose for the remainder of the study. Treatment was discontinued in any patient with two previous dose reductions who had a toxic effect leading to a third dose reduction. The dose levels were as follows: capecitabine (level 1, 1,000 mg/m^2^ bid; level -1, 750 mg/m^2^ bid; level -2, 500 mg/m^2^ bid) and oxaliplatin (level 1, 130 mg/m^2^; level -1, 100 mg/m^2^; level -2, 85 mg/m^2^). Oxaliplatin was discontinued in patients if severe (i.e., grade 2 lasting for >7 days or grade 3) peripheral neuropathy occurred, and these patients then received capecitabine alone according to the same schedule. At the first occurrence of skin toxicity or hand-foot syndrome of grade 2 or more, the capecitabine doses were reduced to level -1 irrespective of the oxaliplatin dose. At the first occurrence of grade 3 neutropenia or grade 3 thrombocytopenia, treatment was held until recovery and then resumed at a full dose. At the second occurrence of grade 3 neutropenia or grade 3 thrombocytopenia, doses of all study drugs were reduced to level 2. At the first occurrence of grade 4 neutropenia or grade 4 thrombocytopenia, doses of all drugs were reduced to level -1. At the second occurrence of grade 4 neutropenia or grade 4 thrombocytopenia, doses of all drugs were reduced to level -2. At the third occurrence, patients were treated outside the protocol.

### Assessment of Efficacy and Toxicity

Enrolled patients underwent complete medical examination at baseline. Assessment of disease extent at baseline was performed with computed tomography of the chest, abdomen, and pelvis. Additionally, follow-up scans were conducted every six weeks during treatment, at the end of treatment or early termination, and every six weeks thereafter until disease progression.

Computed tomography was used to characterize each identified and reported lesion at baseline and during follow-up. The primary endpoint was an intention-to-treat analysis of PFS, measured as time from the date of starting treatment to the date of first documented disease progression or death. Secondary endpoints were overall survival (OS), objective response rate according to RECIST 1.1,^22^ safety, and exploratory biomarker analysis. Response and progression were determined by the local investigator. Adverse events were evaluated at every patient visit based on the Common Terminology Criteria for Adverse Events, version 4.0. Patients were followed up until death or study closure. For exploratory biomarker analysis, we collected archival tumor tissue blocks at initial diagnosis or surgery in enrolled patients, if available.

### Statistical Analysis

Sample size was calculated to reject a PFS of median 8.5 weeks or less in favor of a PFS of median 12 weeks or more with a significance level of 0.1 and power of 90% (H0: 8.5 weeks, H1: 12 weeks, type I error: 10%, and power: 90%). A total of 58 patients were required with an accrual of 20 months and a follow-up of six months after the last patient registry, when an exponential distribution of time to progression was assumed. Allowing a dropout rate of 10%, we aimed to enroll 65 patients. Descriptive statistics were reported as proportions and medians. An event such as PFS or OS was estimated with the Kaplan-Meier method, and the median time-to-event and 95% confidence interval (CI) were estimated. Hazard ratios of XELOX and 95% CIs were estimated using Cox proportional hazards regression. This study was registered at ClinicalTrials.gov (number NCT02350686).

## Results

### Patient Characteristics

From December 2015 to November 2016, 50 patients with metastatic intrahepatic or extrahepatic cholangiocarcinoma or GB cancer were enrolled onto this trial. Patient characteristics are shown in Table 1. The median age of patients was 62.5 years (range, 42-80), and the majority (62%) of patients was male. Thirty-two of 50 patients had received prior surgery with curative intent. Thirty-one patients had well- or moderate-differentiated tumors, and 19 patients exhibited poorly or undifferentiated tumors. The most common metastatic sites were the liver and lymph nodes, followed by the peritoneum. Among 50 patients enrolled onto this study, 13 had a partial response (PR) to first-line GP therapy, and nine had stable disease.

### Toxicity and Dose Delay/Modification

Table 2 shows the toxicity profiles of patients with XELOX. Thrombocytopenia and neuropathy were the most common toxic effects. These side effects were mostly manageable and controllable. For example, hematological toxicity was minimal with only one grade 3/4 neutropenia. The other common adverse events were anorexia, general weakness, nausea, and neutropenia. The grade 3/4 adverse events were as follows: hand-foot syndrome (n=2), neutropenia (n=1), asthenia (n=1), mucositis (n=1), and general weakness (n=1). No patient died of treatment-related causes during the study.

Twenty-six patients received the study drugs without any dose delays. A dose reduction of capecitabine and oxaliplatin was observed in 24 and 23 patients, respectively. The most common cause of dose reduction was thrombocytopenia. Dose modification of capecitabine was required in 18 patients and oxaliplatin in 18 patients. Dose modification of two or more levels was observed in six patients for capecitabine and four patients for oxaliplatin.

### Efficacy and Survival

Intent-to-treat tumor response for all patients enrolled onto this study is shown as Table 3. One complete response (CR) and six partial responses (PRs) were recorded in the XELOX treatment. Nineteen patients had stable disease. The overall response rate and the disease control rate from the intent to treat analysis were 14% and 52%, respectively. Sixteen patients showed disease progression, and eight patients were not eligible for the tumor response.

**Figure 1:**
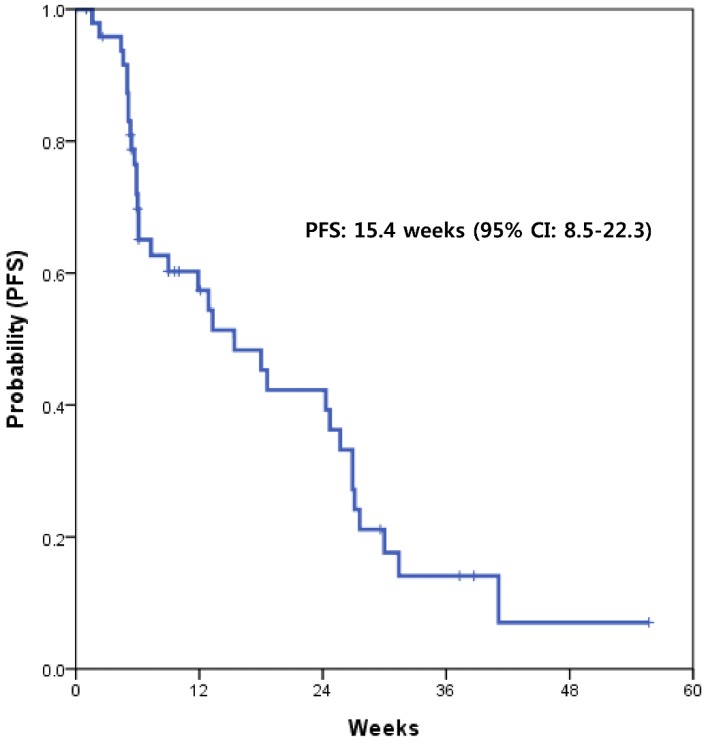
Kaplan-Meier survival curve of progression-free survival (PFS)

**Figure 2:**
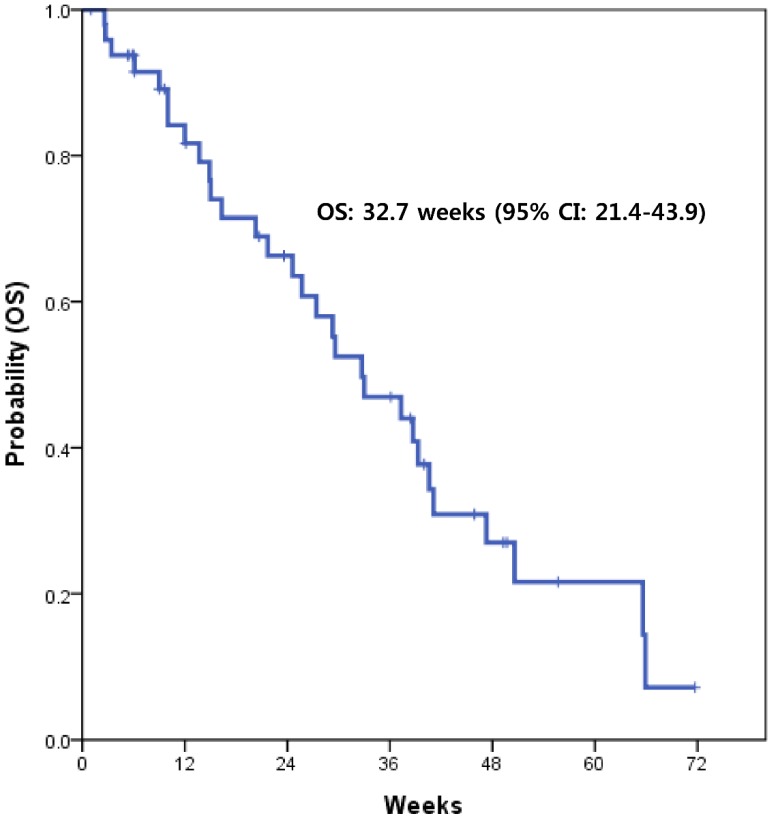
Kaplan-Meier survival curve of overall survival (OS)

With a median follow-up of 15.6 months, the median PFS with XELOX was 15.4 weeks (95% CI, 8.5-22.3) (Fig. 1). This value supported the statistical hypothesis of this study. The median overall survival was 32.7 weeks (95% CI, 21.4-43.9) (Fig. 2).

## Discussion

This prospective phase II trial is the first study to demonstrate the usefulness of capecitabine plus oxaliplatin in patients with advanced BTC who failed first-line treatment of GC. The hypothesis of this study was that XELOX would lead to a median PFS ≥ 12 weeks; a regimen with a median PFS ≥ 12 weeks would warrant further investigation, while study drugs with a median PFS ≤ 8.5 weeks would not be considered as a further study. This study showed a median PFS of 15.4 weeks (95% CI, 8.5-22.3) with XELOX, supporting the hypothesis of this study. Furthermore, this regimen also showed acceptable toxicity profiles compared to those of other studies in the same setting. These findings were achieved using an outpatient schedule.

Following publication of the ABC-02 trial, GC chemotherapy has been established as the standard treatment for advanced BCT.^5^ However, most patients with BTC who receive GC experience progression of the disease. Although BTC has a very aggressive nature, some patients maintain a relatively good performance status after failure to first-line GC. Thus, a tolerable, feasible, and useful second-line treatment is needed for these patients. There have been several studies investigating potential treatments^23^,^24^; however, a standard second-line or salvage treatment has not been established for BTCs. This prospective study suggests that capecitabine plus oxaliplatin might be useful as a second-line therapy in BTCs refractory to the standard GP regimen.

The response rate of capecitabine and oxaliplatin in this study was 14%. In previous studies, the reported response rates were 2-12% for target therapies and 7-10% for salvage cytotoxic chemotherapies.^25^-^28^ Recently, Cereda et al. reported that capecitabine with or without mitomycin had a response rate of 3% in a randomized phase II study of a second-line therapy in patients with BTC.^24^ Considering data from other previous studies, the efficacy of XELOX was slightly better or similar. The disease control rate of XELOX was consistent with previous studies.^23^ In this study, if XELOX showed a median PFS ≥ 12 weeks, we would investigate the study drugs further. The previous randomized phase II trial of second-line therapy in BTC reported a median PFS of 2.1 months. Further, a retrospective analysis of various second-line therapies in BTC showed the median PFS of 3.2 months.^23^ Although there is no standard data of median PFS for the second-line setting in BTCs, we considered a median PFS of 12 or more weeks as the cut-off of usefulness based on the data from these previous studies.^23^,^24^ Herein, we demonstrated that the PFS of XELOX was a median of 15.4 weeks (3.8 months). A prospective randomized study is needed to confirm the usefulness of this regimen in this clinical setting.

Oxaliplatin has replaced the classic cisplatin to reduce emesis and potential renal toxicity without compromising efficacy. 5-FU has extensively been studied in advanced BTCs as a single agent or in combination with platinum compounds. As mentioned, capecitabine is an oral fluoropyrimidine prodrug with preferential conversion to 5-FU in tumor tissue and has shown effective antitumor activity and safety profile in combination with oxaliplatin in various gastrointestinal tumors, including gastric cancer, small bowel cancers, and colon cancer. In advanced BTCs, Nehls et al. revealed the tolerability of XELOX regimen in first line setting.^29^

This regimen showed a mild toxicity profile. The toxicities mainly consisted of grade 1 or 2 events, and thrombocytopenia and neuropathy were the most common adverse events irrespective of grade. These toxicities were easily managed without discontinuation of the study drugs.

BTC is an orphan disease. Because of the rarity and aggressiveness of these tumors and the generally morbid patient population, there is a limited number of prospective studies investigating second-line treatments for advanced BTCs. This phase II trial showed that XELOX was efficacious and had a tolerable toxicity profile. Therefore, XELOX is a promising second-line treatment for patients with BTC.

## Figures and Tables

**Table 1:** Baseline Characteristics of Patients

	Capecitabine plus oxaliplatin (n = 50)
**Age, years**	
Median, range	62·5 (42·0-80·0)
**Sex**	
Male	31 (62%)
Female	19 (38%)
**Primary site**	
Bile ducts	38 (76%)
Gallbladder	12 (24%)
**Previous surgery**	
Yes	32 (64%)
No	18 (36%)
**ECOG performance status**	
0	11 (22%)
1	36 (72%)
2	3 (6%)
**Disease status at study entry**	
Recurrent	25 (50%)
Primarily metastatic	25 (50%)
**Pathologic differentiation**	
Well	11 (22%)
Moderate	20 (40%)
Poorly/Undifferentiated	19 (38%)
**Affected site**	
Liver	29 (58%)
Lymph nodes	29 (58%)
Peritoneum	12 (24%)
Lung	8 (16%)
**Best response to 1^st^ line GP**	
Partial response	13 (26%)
Stable disease	8 (16%)
Progressive disease	29 (58%)

Abbreviations: ECOG, Eastern Cooperative Oncology Group; GP, Gemcitabine plus cisplatin

**Table 2:** Adverse Events

	Capecitabine plus oxaliplatin (n = 50)	
	Grades 1-2	Grades 3-4
Nausea	9 (18%)	
Vomiting	7 (14%)	
Diarrhea	4 (8%)	
Constipation	2 (4%)	
Hand-foot syndrome	5 (10%)	2 (4%)
Anemia	2 (4%)	
Neutropenia	8 (16%)	1 (2%)
Thrombocytopenia	19 (%)	
Elevated AST	1 (2%)	
Skin rash	1 (2%)	
Neuropathy	17 (34%)	
Asthenia	6 (12%)	1 (2%)
Anorexia	10 (20%)	
Mucositis	3 (6%)	1 (2%)
General weakness	10 (20%)	1 (2%)
Neutropenic fever	0	0

Abbreviations: AST, Aspartate Transaminase

**Table 3:** Best Tumor Response According to the Response Evaluation Criteria in Solid Tumors (RECIST) 1·1

	Capecitabine plus oxaliplatin (n = 50)
Overall response rate	7 (14%)
Complete response	1 (2%)
Partial response	6 (12%)
Stable disease	19 (28%)
Progressive disease	16 (32%)
Not evaluable	8 (16%)
